# Ahmed glaucoma valve implantation for congenital ectropion uveae with glaucoma: A case report

**DOI:** 10.1097/MD.0000000000041239

**Published:** 2025-01-17

**Authors:** Fang Sha, Xiujuan Du, Yane Gao, Beibei Wang, Xuan Sun, Kai Tang, Hongsheng Bi

**Affiliations:** a Shandong University of Traditional Chinese Medicine, Jinan, China; b Affiliated Eye Hospital of Shandong University of Traditional Chinese Medicine, Jinan, China; c Shandong Academy of Eye Disease Prevention and Therapy, Jinan, China.

**Keywords:** Ahmed glaucoma valve implantation, congenital ectropion uveae, glaucoma

## Abstract

**Rationale:**

Congenital ectropion uveae (CEU) is a rare, nonprogressive anomaly characterized by the proliferation of the iris pigment epithelium on the anterior surface of the iris, often associated with glaucoma. Due to its rarity and complexity, standardized glaucoma surgical management is limited. To our knowledge, the application of glaucoma drainage devices in CEU is rarely documented. Here, we report a case of Ahmed glaucoma valve (AGV) implantation for unilateral CEU associated with glaucoma.

**Patient concerns:**

A 26-year-old female initially presented with blunt ocular trauma with an intraocular pressure (IOP) of 37 mm Hg in the right eye. After starting glaucoma medication, IOP promptly decreased to 21 mm Hg. However, the patient was subsequently lost to follow-up.

**Diagnoses:**

A definitive diagnosis was not made due to the limited understanding of CEU at the first visit. Nearly 3 years later, the patient was referred to our hospital again with decreased vision for 6 months and mild distending pain in the right eye. The best-corrected visual acuity was 20/25, IOP was 51 mm Hg, and the cup-to-disc ratio was 0.8. Slit-lamp examination of the right eye revealed 360° ectropion uveae, extending around the pupil to the mid-periphery of the iris, which was an unaltered condition since the first visit. Thus, the patient was diagnosed with CEU and unilateral glaucoma.

**Interventions:**

The AGV implantation surgery with mitomycin C was performed in the right eye.

**Outcomes:**

The best-corrected visual acuity of the right eye improved to 20/20. IOP stabilized without medications during the entire period of follow-up for 3 years.

**Lessons:**

Although CEU is rare, ophthalmologists should remain vigilant to avoid missed diagnoses due to its high association with glaucoma. AGV implantation with mitomycin C may be considered an effective surgical management for adult patients with late-onset glaucoma secondary to CEU.

## 1. Introduction

Congenital ectropion uveae (CEU) is a rare ophthalmic disease characterized by uveal ectropion at the pupil margin, iris hypoplasia, iridotrabecular dysgenesis, and is often associated with glaucoma. According to Dowling et al,^[1]^ CEU was first described by Colsman in 1869. Although predominantly unilateral, bilateral cases have also been reported.^[2–5]^ CEU may be associated with other ocular and systemic anomalies. Ocular abnormalities associated with CEU include ptosis, iris coloboma, proptosis, thickened corneal nerves, megalocornea, and Rieger anomaly.^[1,3,6–11]^ Systemic abnormalities include neurofibromatosis, ipsilateral facial hemihypertrophy, and Prader-Willi syndrome.^[4,8,11–13]^

Here, we report a rare case of unilateral CEU associated with glaucoma, unaccompanied by other systemic and ocular associations. It has been reported that all CEU cases became surgical candidates sooner or later.^[5]^ However, there is no consensus on the choice of surgical method, which is chosen according to the experience of the doctor. As far as we know, the use of a glaucoma drainage device (GDD) in CEU is scarce, and this may be the first attempt to perform glaucoma drainage implant surgery in an adult patient presented with unilateral CEU and late-onset glaucoma. Meanwhile, this study reviews the available literature on this rare abnormality.

## 2. Case presentation

**Figure 1.**
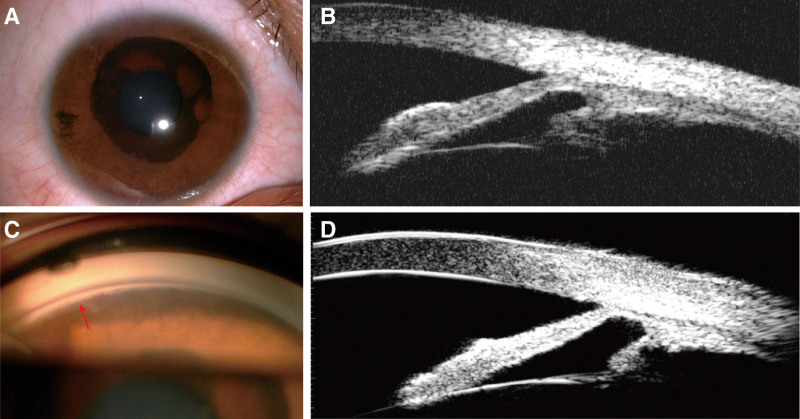
(A and B) The anterior segment photograph showed congenital ectropion uvea, and ultrasound biomicroscopy showed the high anterior insertion of the iris root in the right eye at the first visit in 2018. (C) Gonioscopy examination of the right eye showed an almost closed angle with anterior insertion of the iris, except for the 7 o’clock position with an open angle (red arrow) at the 3-yr follow-up in 2021. (D) Ultrasound biomicroscopy showed an obscured anterior chamber angle with high iris insertion in 2021.

On March 9, 2018, a 26-year-old female was referred to our hospital due to blurred vision in the right eye injured by a wooden stick for 1 hour. On examination, uncorrected visual acuity was 20/25 in the right eye and 20/20 in the left eye, with no improvement in the corrected visual acuity of the right eye. Intraocular pressure (IOP) measured by Goldmann applanation tonometry was 37 mm Hg in the right eye and 19 mm Hg in the left eye. Ocular motility was normal. There was no ptosis in either eye. Slit-lamp examination of the left eye was normal. The right eye showed a clear cornea, a deep anterior chamber, and a round pupil of 3.5 mm, reacting to light. The lenses were clear with intact zonules. Remarkably, slit-lamp examination revealed 360° ectropion uveae, extending around from the pupil to the mid-periphery of the iris, with a sharply demarcated circular border (Fig. 1A). No definitive diagnosis was made due to a lack of understanding of the disease at that time. The patient received topical 2% carteolol twice daily and 1% brinzolamide 3 times daily. Examination after 1 week revealed that visual acuity in the right eye had improved to 20/20, and IOP had returned to 21 mm Hg. One month after the blunt trauma, ultrasound biomicroscopy of the right eye showed the high anterior insertion of the iris root (Fig. 1B). There were no visible abnormalities on fundus examination, but mild visual field loss was observed in the right eye. After that, the patient was lost to follow-up and stopped using antiglaucoma medications.

**Figure 2.**
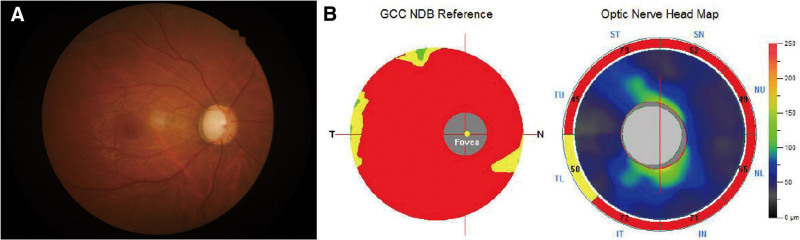
Fundus manifestation of the patient during the second consultation. (A) Optic disc assessment showed advanced glaucomatous disc changes in the right eye. (B) Optical coherence tomography angiography revealed a reduction in the thickness of the peripapillary retinal nerve fiber layer and ganglion cell complex in the right eye.

Nearly 3 years later, the patient again presented to our hospital with the chief complaint of decreased vision in the right eye for 6 months, with mild distending pain recently, on January 11, 2021. She had no evidence of neurofibromatosis, other systemic diseases, or familial diseases. On examination, the left eye was normal, and the right eye was more prominent, with an uncorrected visual acuity of 20/40 and the best-corrected visual acuity of 20/25 with −0.75 diopters spherical and −0.75 diopters cylindrical refractive error. The IOP was 51 and 17 mm Hg in the right and left eyes, respectively. The corneas were clear and equal in diameter, and central corneal thickness was 589 and 583 µm in the right and left eyes, respectively. The right eye had a smooth iris surface without iris crypts or abnormal vessels, and an unaltered iris surface with 360° ectropion uveae since the first visit. However, the pupil was hyporesponsive and had a dilated diameter of 4 mm in the right eye. Gonioscopy examination of the right eye revealed an almost closed angle with anterior insertion of the iris except for the 7 o’clock position, which had an open angle (Fig. 1C). Ultrasound biomicroscopy also showed an obscured anterior chamber angle with high iris insertion (Fig. 1D). The fundus revealed the cup-disc ratio was 0.8 in the right eye with diffuse peripapillary retinal nerve fiber layer loss (Fig. 2A). The thicknesses of the peripapillary retinal nerve fiber layer and ganglion cell complex, as evaluated by optical coherence tomography angiography (Optovue, Inc., Fremont), were reduced in the right eye (Fig. 2B). The visual fields, assessed using the Octopus 900 perimetry (Haag-Streit, Bern, Switzerland), showed asymmetry between the 2 eyes, with tubular vision in the right eye. The final diagnosis was CEU and unilateral glaucoma.

**Figure 3.**
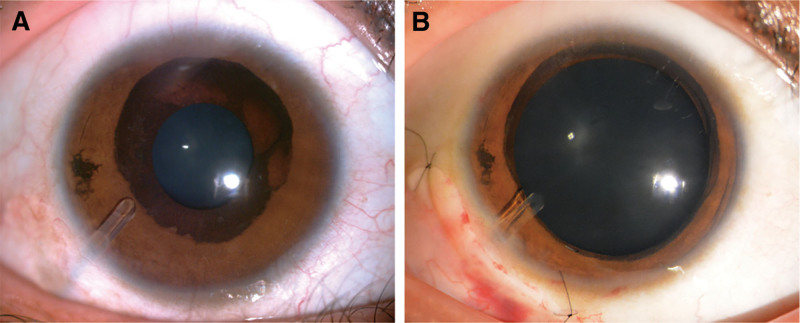
Photograph of the postoperative anterior segment. (A) The anterior segment photograph of the glaucoma drainage valve implanted in the right eye. (B) A narrowing of the everted iris pigment epithelium after mydriasis confirmed the sphincter muscle of the iris and the stroma were not affected in the patient with CEU. CEU = congenital ectropion uveae.

After informed consent, the glaucoma drainage implant, Ahmed FP7 (New World Medical, Inc, Rancho Cucamonga, CA), with mitomycin C (MMC; 0.3 g/L) application for 3 minutes, was performed in the right eye on January 19, 2021. The implantation of the Ahmed glaucoma valve (AGV) was performed as previously described.^[14]^ The patient’s IOP stabilized (ranging from 11 to 13 mm Hg) without medications, and best-corrected visual acuity was 20/20 during the entire period of follow-up for 3 years. The AGV was in place and unobstructed, without proliferation or wrapping of the filtering bleb (Fig. 3A). Not surprisingly, the patient was very satisfied with the result of the treatment.

## 3. Discussion

CEU is a rare congenital disorder resulting from the proliferation of the iris pigment epithelium on the anterior surface of the iris stroma. Ectropion of the iris is believed to be present at birth and nonprogressive,^[1,8,15]^ which is easily differentiated from the acquired variety. The latter results from a fibrovascular membrane on the iris surface and may be associated with iridocorneal endothelial syndrome, neovascular glaucoma, inflammation, or other ischemic or neoplastic factors.^[16–20]^ Moreover, the iris sphincter muscle and stroma are not affected and are not everted on histopathologic examination in CEU, contrary to acquired ectropion uveae (AEU).^[1,21]^ The anterior segment photograph of pupillary dilation in our patient (Fig. 3B) confirms the findings of published reports in the literature.

Apart from the conditions of AEU mentioned earlier, Markovic et al^[22]^ described 3 cases of AEU associated with secondary glaucoma that occurred after blunt trauma to the eye, which had never been reported in the previously published literature. Coincidentally, our patient with a definite history of blunt trauma in the right eye, however, was diagnosed with CEU. In the present case, there were no changes in the shape or size of the pigmented epithelium of the iris on the anterior surface of the iris during the period between the early stage of the eye injury and the diagnosis of CEU after 3 years, which was different from AEU with progressive malformation of the iris. Other typical feature was the anterior insertion of the root of the iris in the case revealed by the gonioscopy examination. Therefore, the accompanying glaucoma in this case was considered to be related to CEU. However, it should be noted that not all cases of CEU exhibit ocular and systemic associations,^[2,23–28]^ as exemplified by the present case.

Glaucoma is a common complication of CEU, ipsilateral to the proliferation of the pigment epithelium, presumably due to severe angle dysgenesis in this disorder. However, the pathogenesis of glaucoma associated with CEU still remains unclear. The previous evidence indicates that developmental abnormalities of the anterior segment in CEU derive from abnormal migration of neural crest cells.^[1,29]^ This theory of the neural crest disorder etiology is reinforced by associations between CEU and neurofibromatosis, Rieger syndrome, as well as prominent corneal nerves.^[3,8,11,12]^ In patients with CEU, Ritch et al^[8]^ found that filmy iris tissue extending to the trabecular meshwork, in a position to obstruct aqueous outflow and cause glaucoma. Harasymowycz et al^[30]^ discovered the presence of a distinct fibrovascular surface membrane covering the anterior aspect of the iris stroma by performing a histopathologic examination of an iris tissue specimen from a patient with CEU during a trabeculectomy (TR) for glaucoma. It was hypothesized that this membrane was responsible for pulling the posterior pigmented layer of the iris anteriorly, creating ectropion. Glaucoma secondary to CEU may present at birth, infancy, or later stages of life, with the majority occurring in the age group of 3 to 15 years, and has been well documented in the literature.^[1,2,5,8,11,13,24,25,31–39]^

Although several reports have described topical therapy for glaucoma secondary to CEU with temporary control of IOP,^[25,32,40]^ the majority of cases of secondary glaucoma are frequently unresponsive to conservative treatment and require surgical interventions in order to achieve long-term IOP control.^[1,4,7–9,12,13,15,23,24,27,30,31]^ However, there is no consensus on the best surgical management for this rare form of glaucoma. A review of different glaucoma surgical procedures performed in patients with CEU is summarized in Table 1. Goniotomy or trabeculotomy is not highly effective in terms of long-term IOP control after surgery, and the need for additional surgery due to severe goniodysgenesis in this disorder.^[1,2,4,8,11]^ Mandal and Gothwal^5]^ reported a retrospective chart review of 26 eyes in 21 patients with CEU to evaluate the long-term outcomes of glaucoma management, with a median follow-up of 51.1 months. They concluded that combined trabeculotomy-trabeculectomy (CTT) is a safe and efficacious primary procedure for the management of early-onset glaucoma in CEU, and TR with or without adjuvant MMC is a viable second-line treatment in late-onset glaucoma with CEU. In the cases published by Singh et al,^[27]^ Kumari and Saha,^[9]^ and Seymenoğlu and Başer,^[24]^ patients with CEU subjected to CTT with antifibrotics (0.02% MMC) obtained a good control of IOP, which suggested that early surgical intervention is required, as explained in their case reports. In a study described by Kaushik et al,^[2]^ 20 eyes of 10 infants with neonatal-onset CEU were compared with 16 eyes of 9 patients with neonatal-onset primary congenital glaucoma. All patients underwent goniotomy or trabeculotomy, with TR depending on corneal clarity. Thirteen of the 16 (81.2%) eyes with neonatal-onset primary congenital glaucoma had a good or satisfactory outcome compared with 6 of the 20 (30%) eyes with neonatal-onset CEU. In addition, the results also showed that glaucoma secondary to CEU may require coupling with additional surgeries to achieve IOP control, which has been supported in the recent reports.^[4,6,7,13]^

**Table 1:** Summary of published studies on clinical findings and management of glaucoma in patients with CEU.

Authors	Year	Number of patients	Gender	Age range	Glaucoma (number of eyes)	Other manifestations	Management	Follow-up
Quigley and Stanish^[32]^	1978	1	M	41 yr	1 (100%)	NA	TT	2 yr
Gramer and Krieglstein^[35]^	1979	2	1 (50%) M 1 (50%) F	1 yr and 3 yr	2 (100%)	NA	1 (50%) GO × 2 1 (50%) GO, CCT	NA
Ritch et al^[8]^	1984	8	5 (62.5%) M 3 (37.5%) F	10 wk to 31 yr	7 (87.5%)	3 (37.5%) neurofibromatosis, 2 (25%) facial hemihypertrophy, 1 (12.5%) Prader-Willi syndrome, 1 (12.5%) Rieger anomaly, 1 (12.5%) ptosis	2 (25%) TT, TR, ALT × 2.1 (12.5%) TT, TR × 2. 1 (12.5%) GO, TR 2 (25%) TT, ALT 1 (12.5%) TR	NA
Dowling et al^[1]^	1985	10	8 (80%) M 2 (20%) F	4.5 mo to 42 yr	9 (90%)	6 (60%) ptosis	1 (10%) TT, GO × 2, TO, E. 2 (20%) TT, TR 1 (10%) TT, 2 TR 1 (10%) TT, GO, PS 1 (10%) GO, TR × 2 1 (10%) 2 GO, 2 PS 2 (20%) TT	4 mo to 19 yr
Hertzberg^[36]^	1985	1 (2 eyes)	M	4 yr	2 (100%)	Ptosis, asthma, dental defect	2 (100%) TR	18 yr
Futterweit et al^[12]^	1986	1	M	8 mo	1 (100%)	Prader-Willi syndrome	TT, ALT, TR	NA
Khorram and Mets^[37]^	1997	1	M	6 yr	1 (100%)	No other manifestations	TO	NA
Dietlein et al^[38]^	1998	1	M	15 yr	1 (100%)	Vitreoretinal degeneration, neurofibromatosis, ptosis	TT, TR with MMC	NA
Mandal^[5]^	1999	1	F	9 yr	1 (100%)	Thickened corneal nerves	CTT	NA
Bansal and Luck^[34]^	2002	2	1 (50%) M 1 (50%) F	15 and 5.5 yr	2 (100%)	1 (50%) neurofibromatosis, ptosis	1 (50%) TT, TR 1 (50%) CTT	NA
Sethi et al^[3]^	2005	1 (2 eyes)	M	22 yr	2 (100%)	Thickened corneal nerves	1 (100%) TR with MMC	3 mo
Harasymowycz et al^[30]^	2006	1	F	3 yr	1 (100%)	NA	1 (100%) TT, TR with MMC	3 mo
Grieshaber et al^[39]^	2006	1	M	5 yr	1 (100%)	Intestinal neuronal dysplasia	1 (100%) PS, TO	3 mo
Monaco et al^[23]^	2009	1	F	6 yr	1 (100%)	No other manifestations	1 (100%) TT, TR	2 yr
Seymenoğlu and Başer^[24]^	2011	1 (2 eyes)	F	9 yr	2 (100%)	No other manifestations	2 (100%) TT, TR with MMC	4 yr
Edward et al^[11]^	2012	5	2 (40%) M 3 (60%) F	Birth to 13 yr	5 (100%)	5 (100%) neurofibromatosis, 4 (80%) megalocornea, and buphthalmos	1 (20%) TO, TR with MMC, GDD, E 1 (20%) TR with MMC, E. 1 (20%) TR with MMC, CPC × 2, E. 2 (40%) E	NA
Laaks and Freeman^[26]^	2013	1	M	5 mo	1 (100%)	No other manifestations	TT, TO	1 yr
Ziakas et al^[25]^	2014	1	M	3 yr	1 (100%)	No other manifestations	TT	1 yr
Li et al^[41]^	2015	1 (2 eyes)	M	1 mo	1 (50%)	Neurofibromatosis	1 (50%) TR with MMC, GDD	NA
Kumari and Saha^[9]^	2018	1	F	12 yr	1 (100%)	Ptosis and proptosis	TT, TR with MMC	3 mo
Wang et al^[13]^	2018	3	1 (33.3%) M 2 (66.7%) F	7, 8 and 13 yr	3 (100%)	1 (33.3%) facial hemihypertrophy, 2 (66.7%) ptosis	1 (33.3%) TT, TO, TR, GDD, CPC 1 (33.3%) TT, GDD, CPC 1 (33.3%) TT, GDD, TR with MMC	3–11 yr
Hatami et al^[7]^	2019	1	M	7 yr	1 (100%)	Hemifacial, hypertrophy, ptosis, and proptosis	TT, TR, GDD	1 yr
Lim and Ho^[31]^	2020	3	1 (33.3%) M 2 (66.7%) F	3, 5, and 30 yr	3 (100%)	3 (100%) ptosis	3 (100%) TT, TR with MMC	5–15 yr
Venkataraman et al^[40]^	2020	1	F	9 yr	1 (100%)	Neurofibromatosis	TT	1 mo
Kaushik et al^[2]^	2021	10 (20 eyes)	NA	15.3–59.3 d	20 (100%)	No other manifestations	2 (10%) GO 18 (90%) TR	1 yr
Singh et al^[27]^	2021	1	M	10 yr	1 (100%)	No other manifestations	1 (100%) TT, CTT	7 mo
Jacobson et al^[4]^	2022	8 (11 eyes)	2 (25%) M 6 (75%) F	0.01–14.1 yr	11 (100%)	1 (12.5%) Hemifacial Hypertrophy, 4 (50%) ptosis	1 (9%) TT, TR, GDD, CPC 1 (9%) TT, GDD, CPC 1 (9%) TT, GDD, TR with MMC, GDD removal, GDD. 1 (9%) TT, TO 1 (9%) TT, TR with MMC. 4 (36%) TT, TO, GDD 2 (18%) TT, TO, TR with MMC	0.3–19 yr
Snehi et al^[6]^	2022	5	2 (40%) M 3 (60%) F	7–17 yr	5 (100%)	5 (100%) ptosis, myopia	3 (60%) TR with MMC 1 (20%) GO, TR with MMC. 1 (20%) TR with MMC, GDD	18–82 mo
Mandal and Gothwal^[5]^	2023	21 (26 eyes)	10 (48%) M 11 (52%) F	6 d to 19 yr	26 (100%)	NA	17 (65%) CTT 3 (12%) TR with MMC 2 (8%) TR 3 (12%) CPC 1 (4%) EV	51.1 yr
Incandela et al^[28]^	2023	1	F	18 yr	1 (100%)	No other manifestations	TT, ALT	1 yr

ALT = argon laser trabeculoplasty, CCT = cyclocryotherapy, CEU = congenital ectropion uveae, CPC = cyclophotocoagulation, CTT = combined trabeculotomy-trabeculectomy, E = enucleation, EV = evisceration, F = female, GDD = glaucoma drainage device, GO = Goniotomy, M = male, MMC = mitomycin C, NA = not available, PS = posterior sclerectomy, TO = trabeculotomy, TR = trabeculectomy, TT = topical therapy.

In the literature, all CEU cases became surgical candidates sooner or later, however, the surgical option was determined according to the clinical experience of the surgeons, as reported by Mandal and Gothwal^[5]^ To our knowledge, the use of GDDs in CEU is relatively sparse compared with angle surgery, TR, and CTT in published reports. In a retrospective interventional case series published by Jacobson et al,^[4]^ 7 eyes of 5 patients (infants or children) with CEU underwent GDD placement, of which 3 required additional surgery. Wang et al,^[13]^ from the same team as Jacobson, described 3 cases of angle-closure glaucoma in children with CEU had early GDD encapsulation (within 4 months) and required additional surgery (cycloablation or TR). Moreover, only several cases of children with CEU underwent GDD following the failure of MMC-augmented TR to achieve good IOP control.^[6,7,41]^

The present case is distinctive in that we performed AGV as the initial treatment in an adult with CEU in late-onset glaucoma, based on our experience and the results of AGV in various forms of refractory glaucoma previously reported.^[14,42–44]^ Meanwhile, we applied intraoperative MMC to the eye, whereas other previous cases were without antimetabolites on a GDD procedure in children with CEU.^[4,6,7,13,41]^ In refractory glaucoma, shunt procedures with antifibrotics have been a preferred surgical management.^[42,45]^ As expected, IOP control was obtained with a single procedure of MMC-augmented AGV in the present study. No serious intraoperative or postoperative complications were noted. However, the final surgical outcome still requires long-term follow-up.

In conclusion, CEU is a rare disease often associated with progressive glaucoma, resistant to medical therapy. Clinicians should be aware of patients presenting with congenital ectropion of the posterior pigment epithelium of the iris, who should be carefully examined periodically to detect glaucoma. So far, there is no consensus on the choice of surgical method for this rare form of glaucoma. AVG with MMC, as a preferred surgical management in this report, was effective in lowering IOP in CEU with late-onset glaucoma, which requires further validation with long-term follow-up.

## Author contributions

**Data curation:** Fang Sha, Xuan Sun.

**Project administration:** Fang Sha, Beibei Wang, Kai Tang.

**Writing – original draft:** Fang Sha, Xiujuan Du.

**Investigation:** Xiujuan Du, Kai Tang.

**Visualization:** Yane Gao, Xuan Sun.

**Writing – review & editing:** Yane Gao, Beibei Wang, Hongsheng Bi.

**Conceptualization:** Hongsheng Bi.
